# Prediction of Prognosis and Immunotherapy of Osteosarcoma Based on Necroptosis-Related lncRNAs

**DOI:** 10.3389/fgene.2022.917935

**Published:** 2022-05-26

**Authors:** Guowei Wang, Xiaobo Zhang, Wanjiang Feng, Jianlong Wang

**Affiliations:** Department of Orthopedics, Third Xiangya Hospital, Central South University, Changsha, China

**Keywords:** osteosarcoma, necroptosis, lncRNA, bioinformatics, prognosis

## Abstract

**Background:** Osteosarcoma (OS) is the most common primary tumor of bone in adolescents, and its survival rate is generally less than 20% when metastases occur. Necroptosis, a novel form of programmed necrotic cell death distinct from apoptosis, has been increasingly recognized as a promising therapeutic strategy. This study sought to identify long non-coding RNAs (lncRNAs) associated with necrotizing apoptosis to predict prognosis and target drug use to improve patient survival.

**Methods:** Transcriptomic data and clinical data from 85 OS patients with survival time data and expression profiles from 85 random normal adipose tissue samples were extracted from the UCSC Xena website (http://xena.ucsc.edu/). Nine necroptosis-associated differential prognostic lncRNAs were then identified by analysis of variance, correlation analysis, univariate Cox (uni-Cox) regression, and Kaplan–Meier analysis. Then, patients were randomized into training or testing groups. According to uni-Cox, we obtained prognostic lncRNAs in the training group and intersected them with the abovementioned nine lncRNAs to obtain the final necrotizing apoptosis–related differential prognostic lncRNAs (NRlncRNAs). Next, we performed the least absolute shrinkage and selection operator (LASSO) to construct a risk model of NRlncRNAs. Kaplan–Meier analysis, ROC curves, nomograms, calibration curves, and PCA were used to validate and evaluate the models and grouping. We also analyzed the differences in tumor immunity and drugs between risk groups.

**Results:** We constructed a model containing three NRlncRNAs (AL391121.1, AL354919.2, and AP000851.2) and validated its prognostic predictive power. The value of the AUC curve of 1-, 3-, and 5-year survival probability was 0.806, 0.728, and 0.731, respectively. Moreover, we found that the overall survival time of patients in the high-risk group was shorter than that in the low-risk group. GSEA and ssGSEA showed that immune-related pathways were mainly abundant in the low-risk group. We also validated the differential prediction of immune checkpoint expression, tumor immunity, and therapeutic compounds in the two risk groups.

**Conclusion:** Overall, NRlncRNAs have important functions in OS, and these three NRlncRNAs can predict the prognosis of OS and provide guidance for immunotherapy in OS.

## Introduction

Osteosarcoma (OS) is the most common primary malignant tumor of bone in adolescents. Currently, treatment for osteosarcoma is mainly neoadjuvant chemotherapy and surgery (Gill and Gorlick, 2021). Over the past three decades, limited progress has been made in improving survival outcomes for patients with osteosarcoma. Particularly, the survival rate of patients with metastasis is only 20% (Meltzer and Helman, 2021). Therefore, it is crucial to search for a liable and specific biomarker for the diagnosis and prognosis of OS.

Long non-coding RNAs (lncRNAs) are a kind of transcriptional RNAs with a length of more than 200 nucleotides and are not translated into proteins (Bridges et al., 2021). The expression and mutations of lncRNAs can affect the occurrence and metastasis of tumors. The functions of lncRNAs may be to inhibit or promote carcinogenic processes (Bhan et al., 2017). McCabe et al. reported that lncRNAs can affect cancer stem cell function and epithelial–mesenchymal transition (McCabe and Rasmussen, 2021). Moreover, lncRNAs can regulate the transcription and translation of metabolism-related genes or the modification of metabolism-related proteins to influence energy metabolism and cancer progression (Tan et al., 2021). Zhang et al. described the mechanism of lncRNA resistance to chemotherapy and radiotherapy (Xinyi Zhang et al., 2020). Thus, lncRNAs can be used as biomarkers of cancer progression and potential therapeutic targets.

Necroptosis, a novel form of programmed necrotic cell death distinct from apoptosis, is dependent on receptor interacting protein kinase 1/3 (RIPK1/RIPK3) and mixed lineage kinase domain-like pseudokinase (MLKL) (Galluzzi et al., 2017). In recent years, necroptosis has been suggested to play a major role in tumor regulation, and targeting necroptosis has been suggested as a potential tool for novel cancer therapies (Gong et al., 2019; Yan et al., 2022). The complex role of necroptosis in tumor progression, tumor metastasis, tumor prognosis, tumor immune regulation, tumor subtype determination, and tumor therapy has been summarized by Gong et al (2019). However, the mechanism of the role of necroptosis in tumor regulation is unclear, and studies on the role of necroptosis-associated lncRNAs in OS are inconclusive.

In this study, we analyzed the expression of lncRNAs in OS and normal adipose tissue from the UCSC Xena website and screened for necrotizing apoptosis–related lncRNAs.

## Materials and METHODS

### Data Collection

RNA-sequencing (RNA-seq) data and clinical features were obtained from the UCSC Xena website (http://xena.ucsc.edu/) on 1 April 2022, including 85 tumor datasets and 85 random adipose tissue datasets (Chandrashekar et al., 2017). Data for 67 necroptosis-associated genes were obtained from a previous report (Zhao et al., 2021).

### Screening and Differential Expression Analysis of Necrotizing Apoptosis–Related lncRNAs

The packages of “limma” (Wettenhall and Smyth, 2004) and “igraph” were used to plot the Sankey relationship between necroptosis genes and necroptosis-associated lncRNAs by Pearson’s correlation analysis (|Pearson R| >0.4 and *p* < 0.001). The Kaplan–Meier analysis and univariate Cox regression analysis (uni-Cox) were used to select necroptosis-associated lncRNAs with prognostic relevance. Differentially expressed lncRNAs were explored by the package “limma”. Differential necroptosis-related prognostic lncRNAs were mapped by the package “pheatmap”.

### Risk Modeling

To investigate the prognostic sensitivity of prognostic necrotizing apoptosis–associated lncRNAs using expression and clinical data from the UCSC Xena website, Kaplan–Meier analysis, univariate Cox regression analysis, correlation analysis, and differential expression analysis were used to screen for nine prognostically relevant necroptosis-related lncRNAs (*p* < 0.05). Next, we randomly divided all samples into training and testing groups in a ratio of 8:2 and performed univariate Cox regression analysis in the training group to screen out seven of the abovementioned nine lncRNAs. To prevent overfitting, we used the least absolute shrinkage and selection operator (LASSO) regression analysis risk score = Ʃ [Exp (lncRNA) × coef (lncRNA)] and ran 1000 cycles to create the final predictive model for necroptosis-associated lncRNAs (NRlncRNAs) (Bunea et al., 2011). We divided all samples into two groups based on median risk scores: the high-risk group and low-risk group. We used principal component analysis (PCA) to validate the credibility of the model. In addition, the predictive power of the model was evaluated by receiver operating characteristic (ROC) analysis. Kaplan–Meier curve analysis was performed to assess the significance of overall survival differences between high-risk and low-risk groups.

### Independent Prognostic Analysis

We explored whether clinical characteristics (age, gender, and tumor metastasis) could be used as independent prognostic factors by using univariate and multivariate independent prognostic analyses of Cox regression.

### Construction of the Nomogram

We used clinical factors (age, gender, and tumor metastasis) and the risk score of our model to build a prognostic nomogram to predict 1-, 3-, and 5-year overall survival in OS patients. The model was calibrated by the calibration plot.

### Functional Analysis

We investigated the enrichment analysis of the Kyoto Encyclopedia of Genes and Genomes (KEGG) in high-risk and low-risk populations by packages “enrichplot” and “clusterProfiler” (Damian and Gorfine, 2004; Yu et al., 2012).

### Tumor Immune Assessment

We used the CIBERSORT algorithm to investigate the relationship between immune cells and risk groups and predict the correlation between risk scores and immune cells (Newman et al., 2015). Differential expression of immune checkpoints and tumor microenvironment (TME) scores (including ESTIMATE scores, stromal scores, and immune scores) between risk groups was also investigated.

### Predicting Potential Compounds for the Treatment of Osteosarcoma

In order to predict potential drugs that could be used for OS treatment, we calculated IC_50_ values for drugs obtained from the GDSC website (https://www.cancerrxgene.org/). The therapeutic effect of drugs on high-risk and low-risk groups was explored by the package “pRRophetic” (Geeleher et al., 2014).

## Results

### Prognosis-Related lncRNAs Co-expressed With Necroptosis

**FIGURE 1:**
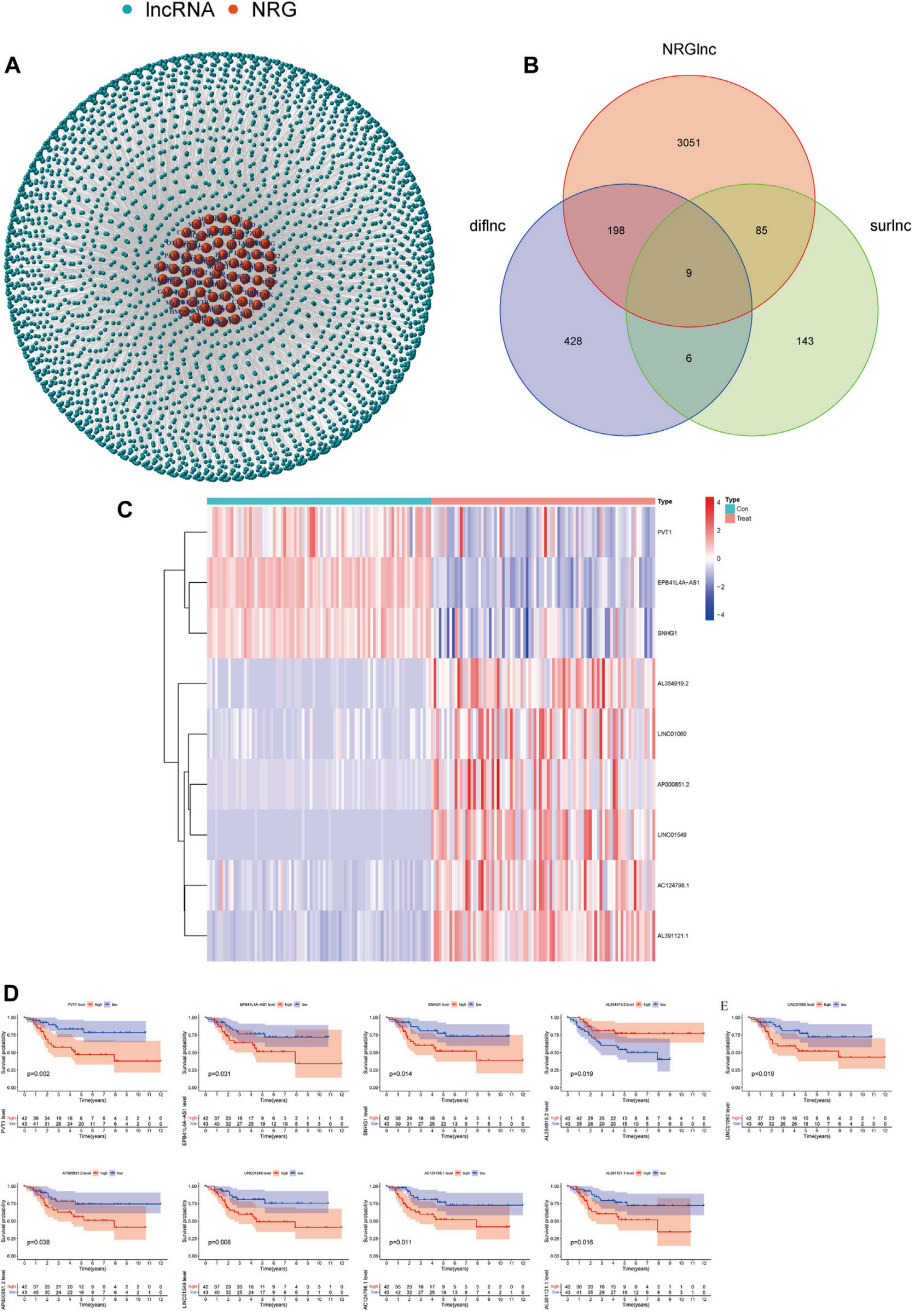
Screening of necroptosis-related lncRNAs. **(A)** Necroptosis-related lncRNA–mRNA co-expression network diagram. **(B)** Venn diagram of necroptosis-related lncRNA, prognosis-related lncRNAs, and differential lncRNAs. **(C)** Expression profiles of nine prognostic lncRNAs. **(D)** Survival curve of nine prognostic lncRNAs.

We identified 3,343 lncRNAs (|Coefficient| > 0.4 and *p* < 0.001) that were co-expressed in OS (Figure 1A). By Kaplan–Meier analysis and univariate Cox analysis, 243 prognosis-related lncRNAs (surlnc) were identified (*p* < 0.05) (Supplementary Table S1). We obtained 641 differential lncRNAs (diflnc) (|Log ₂ FC| > 1 and *p* < 0.05) by differential analysis of combining 85 patients with OS and 85 random normal adipose tissue samples in GTEx (Supplementary Table S2). Then, nine differentially expressed prognostic lncRNAs for necroptosis were identified by taking the intersection of the aforementioned three sets (Figure 1B). We drew their pheatmap and survival curve by packages of “pheatmap” and “survival” (van Dijk et al., 2008) (Figures 1C, D).

### Model Construction and Validation

**FIGURE 2:**
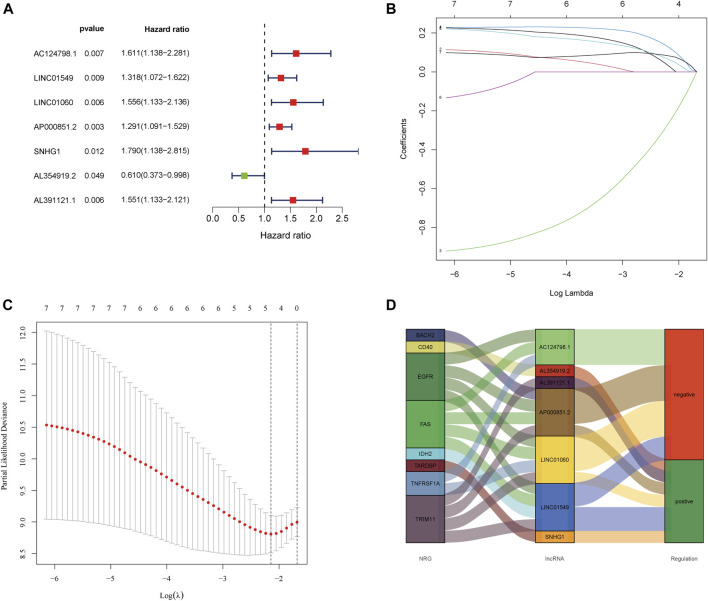
Extraction of the prognostic signature of final necroptosis-related lncRNAs. **(A)** Univariate Cox regression analysis of prognostic lncRNAs in the training group. **(B)** LASSO coefficient profiles of necroptosis-related lncRNAs in the training group. **(C)** Partial likelihood deviance of prognostic signature. **(D)** Sankey diagram of necroptosis genes and related lncRNAs in the training group.

First, 85 tumor samples were randomly divided into two groups in a ratio of 8:2. We constructed a univariate Cox regression analysis in the training group and further found that seven of the aforementioned nine lncRNAs were associated with prognosis (Figure 2A). Subsequently, a model was constructed by performing LASSO regression analysis to predict OS prognosis (Figures 2B,C). Then, three lncRNAs (AL391121.1, AL354919.2, and AP000851.2) were identified. The risk score was calculated as follows: risk score = (0.316737556966157 * AL391121.1 exp.) + (-0.905740574364951 * AL354919.2 exp.) + (0.205992179621899 * AP000851.2 exp.). The sample was divided into high-risk and low-risk groups based on the median risk score. We could find that AL391121.1 and AL354919.2 were positively regulated by the necroptosis gene in the Sankey diagram of the training group (Figure 2D).

**FIGURE 3:**
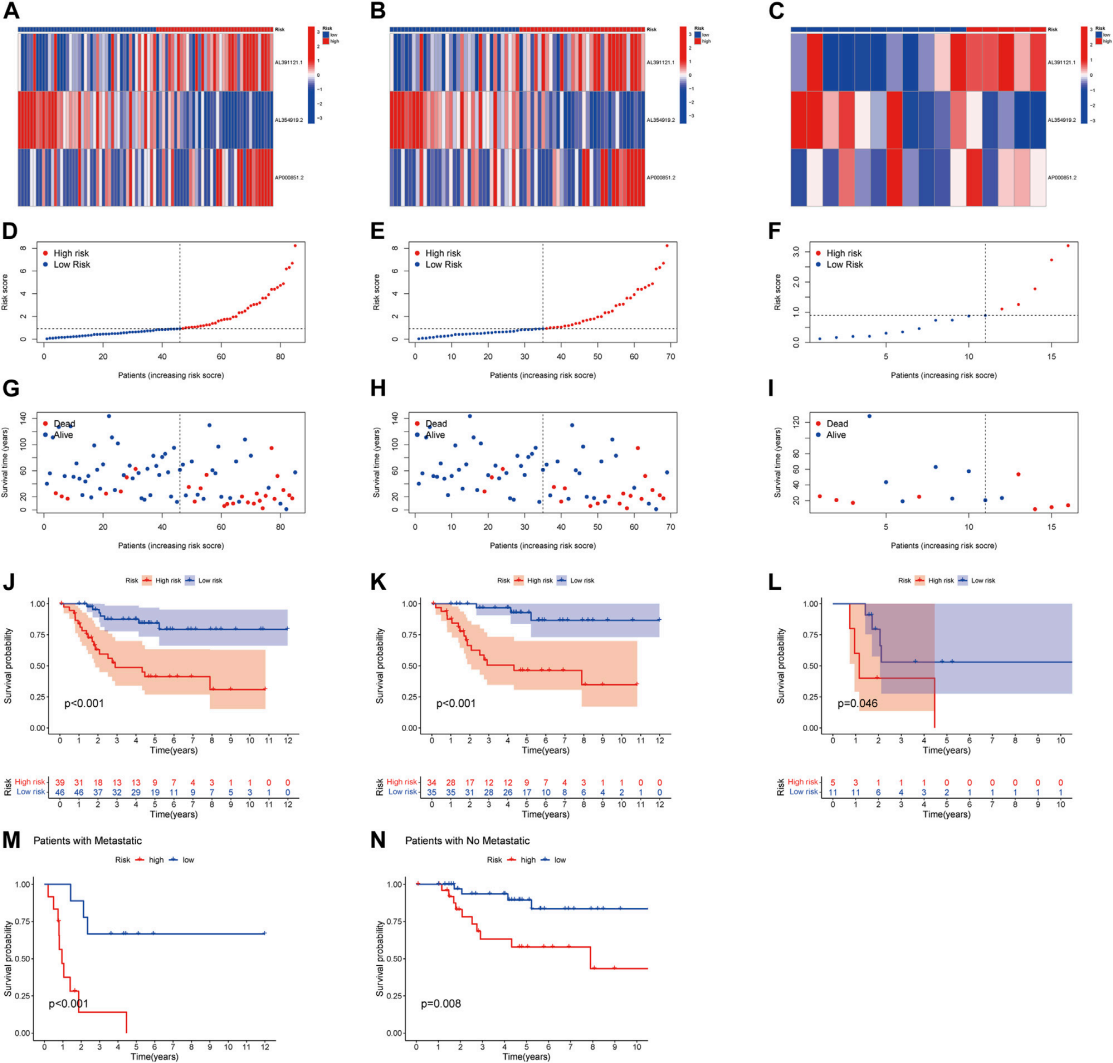
Prognosis of the risk model in the entire, training, and testing sets. **(A–C)** Heatmap of the expression of three lncRNAs in the entire, training, and testing sets, respectively. **(D–F)** Risk model of the entire, training, and testing sets, respectively. **(G–I)** Survival time and survival status in the entire, training, and testing sets, respectively. **(J–L)** Kaplan–Meier survival curves of patients with OS in the entire, training, and testing sets, respectively. **(M–N)** Kaplan–Meier survival curves of patients with tumor metastasis and tumor non-metastasis.

To assess the prognostic power of the risk model, we compared the correlated expression, distribution of risk scores, and survival status of the three NRlncRNAs between risk groups in the whole group, training group, and test group (Figures 3A–L). Obviously, the lower the risk score, the higher the survival rate. According to the survival analysis, the prognosis of the low-risk group was better than that of the high-risk group, with a statistically significant difference. Similarly, this result was validated in the tumor metastasis group or tumor non-metastasis group (Figures 3M, N).

**FIGURE 4:**
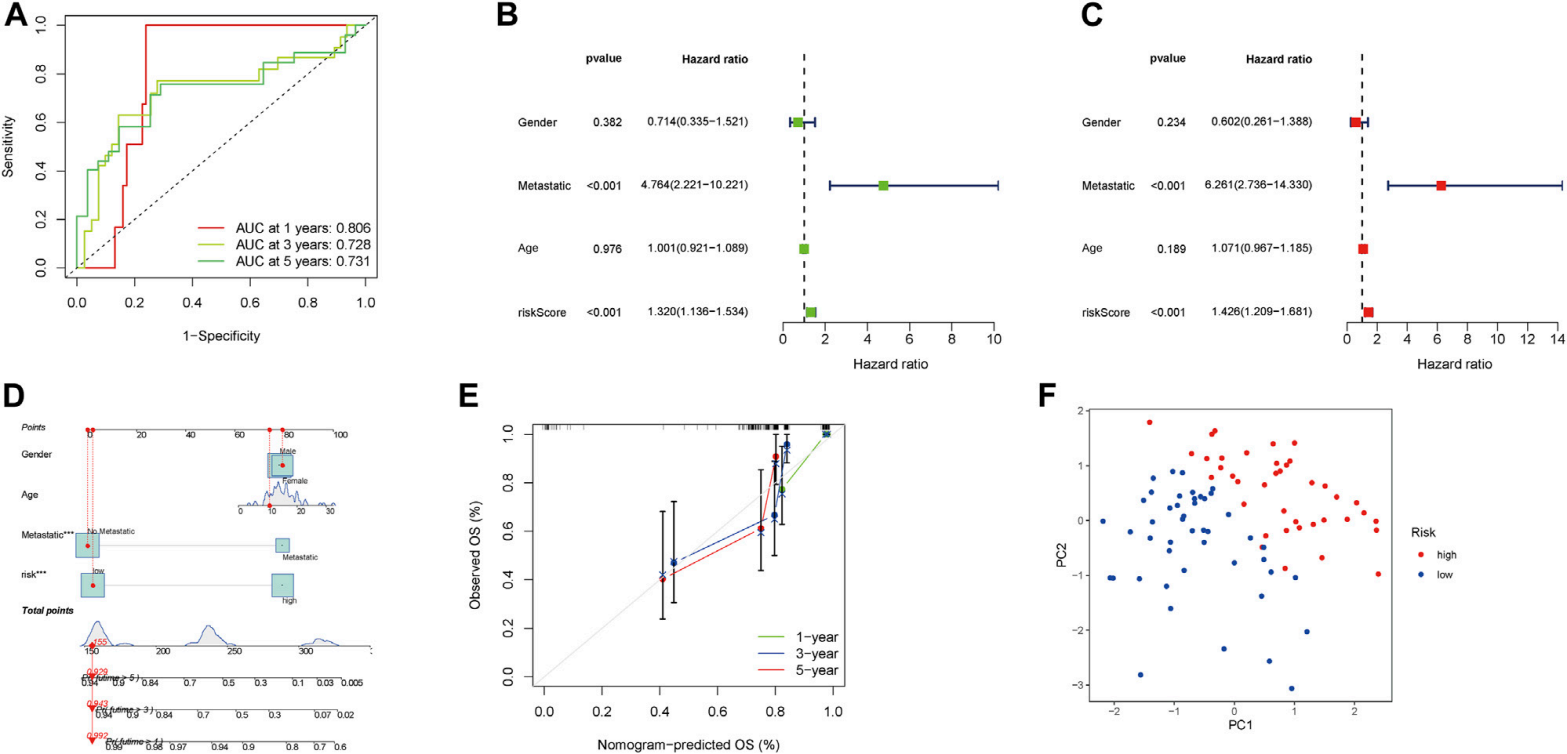
Assessment and nomogram of the necroptosis-related lncRNA signature. **(A)** 1-, 3-, and 5-year ROC curves of the entire sets. **(B–C)** Uni-Cox and multi-Cox analyses of clinicopathologic factors and risk score with overall survival. **(D)** Nomogram for predicting overall survival. **€** 1-, 3-, and 5-year overall survival of calibration curves. **(F)** PCA scatterplot of the sample distribution.

The AUCs of 1-, 3-, and 5-year survival were 0.806, 0.728, and 0.731, respectively, which indicated the NRlncRNA signature is a good predictive value (Figure 4A). The hazard ratio (HR) and 95% confidence intervals (CI) for the risk scores were 1.320 and 1.136–1.534 for the uni-Cox regressions (*p* < 0.001), and 1.426 and 1.209–1.681 for the multi-Cox regressions (*p* < 0.001), respectively. In addition, we identified metastasis as an independent prognostic factor by uni-Cox regressions (HR = 4.764, CI = 2.221–10.221, *p* < 0.001) and multi-Cox (HR = 6.261, CI = 2.736–14.330, *p* < 0.001) (Figures 4B,C). Also constructed were clinical-pathological variables and risk scores to predict the prognosis of OS patients at 1, 3, and 5 years (Figure 4D). The calibration curves showed good agreement between actual overall survival rates and predicted survival rates at 1, 3, and 5 years (Figure 4E). In addition, principal component analysis (PCA) showed that patients with different risks were divided into two clusters (Figure 4F).

### GSEA Enrichment Analysis

**FIGURE 5:**
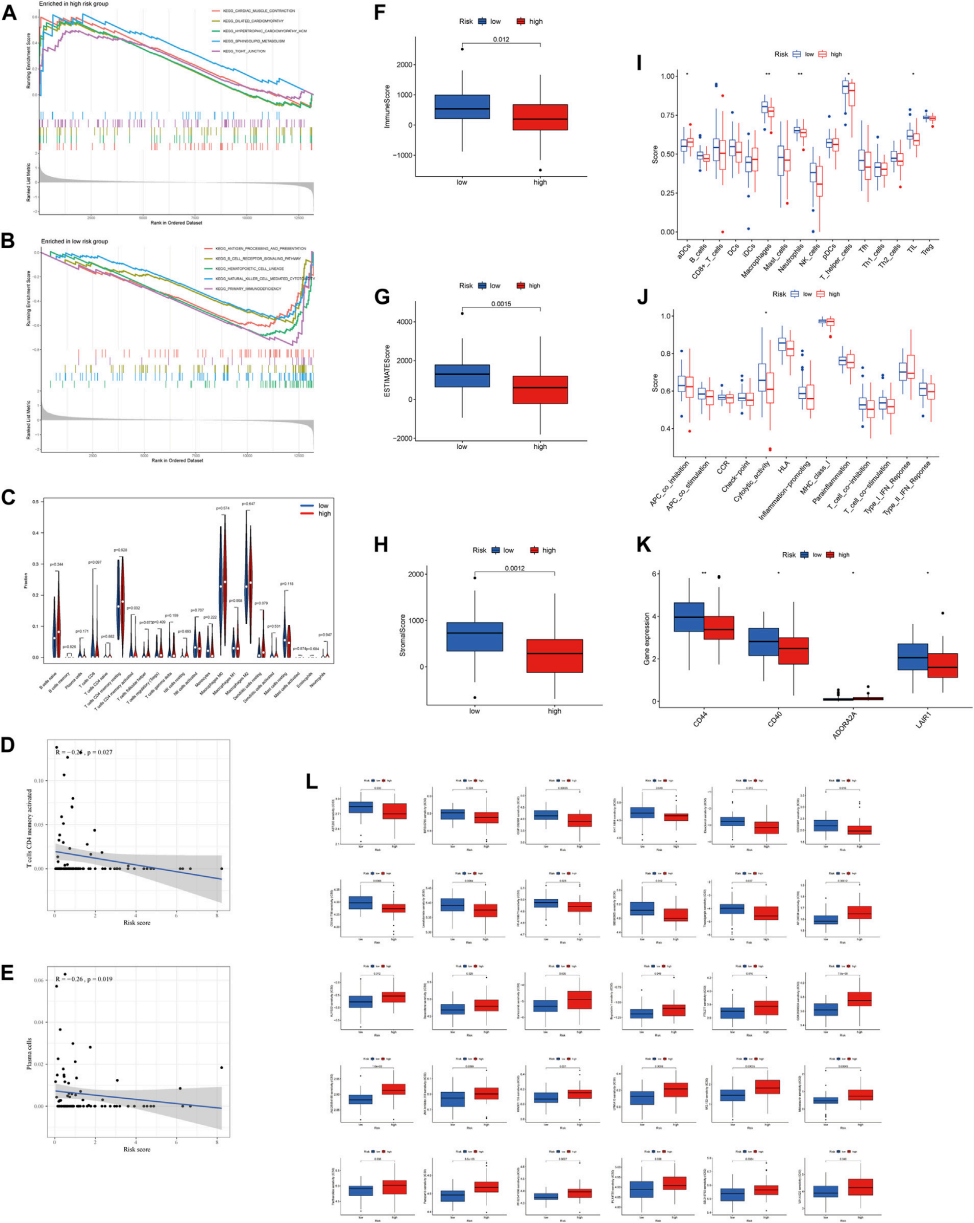
Immune signature in the high-risk and low-risk groups. **(A, B)** GSEA analysis in the high-risk and low-risk groups, respectively. **(C)** Expression of immune cells between two groups. **(D)** Relationship between risk score and T cell CD4 memory activation. **(E)** Relationship between risk score and plasma cells. **(F–H)** Differential expression of TME scores (immune scores, ESTIMATE scores, and stromal scores) between risk groups. **(I)** Differential expression of cell infiltration between risk groups is based on the ssGSEA scores. **(J)** Immune functional differences between risk groups are based on the ssGSEA scores. **(K)** Differential expression of immune checkpoints between risk groups. **(L)** Sensitivity performance of 30 drugs in the high-risk and low-risk groups.

GSEA was performed for KEGG pathway enrichment analysis to clarify the differences of enrichment pathways between low-risk and high-risk groups. The results showed that a number of cancer and metabolism-related pathways were enriched, including the sphingolipid metabolism signaling pathway and tight junction signaling pathway, which were significantly associated with the high-risk group (Figure 5A). In the low-risk group, the primary immunodeficiency signaling pathway, hematopoietic cell lineage signaling pathway, B cell receptor signaling pathway, antigen processing and presentation signaling pathway, and natural killer cell–mediated cytotoxicity signaling pathway were significantly enriched (Figure 5B). Therefore, we hypothesized that necroptosis may be involved in the occurrence and development of OS through immune-related pathways.

### Investigation of Immunological Factors and Drug Efficacy in the High-Risk Group

Differential expression of 22 immune cell infiltration fractions [naive B cells, memory B cells, plasma cells, CD8 T cells, naive CD4 T cells, memory resting CD4 T cells, memory activated CD4 T cells, T follicular helper cells, T cells regulation (Tregs), gamma-delta T cells, resting NK cells, activated NK cells, monocytes, macrophages M0, macrophages M1, macrophages M2, resting dendritic cells, activated dendritic cells, resting mast cells, activated mast cells, eosinophils, and neutrophils] showed that T cell CD4 memory activation was significantly different between the high-risk group and low-risk group (Figure 5C). In addition, we found that lower risk scores correlated more with T cell CD4 memory activation and plasma cells (Figures 5D,E). All of these suggest that the low-risk group has a higher immune infiltration status. Furthermore, we found that the low-risk group had a higher immune score, ESTIMATE score, and stromal score (Figures 5F–H). Subsequently, ssGSEA was performed to assess the level of immune cell infiltration (*p* < 0.05). The results showed that the infiltration level of aDCs, macrophages, neutrophils, T helper cells, and TIL was significantly different between risk groups (Figure 5I). Moreover, the relationship between the risk score and immune pathways in OS was investigated. The boxplot of the results showed that cytolytic activity was correlated with risk score (Figure 5J).

The expression of immune checkpoints (CD44, CD40, and LAIR1) was higher in the low-risk group than in the high-risk group, while the opposite result was observed for ADORA2A (Figure 5K). In addition, drug sensitivity analysis was performed between risk groups by the “pRRophetic” algorithm to find the best therapeutic drugs for different risk groups. The IC_50_ of the 30 drugs applied to the treatment of OS treatment was different between the high-risk group and low-risk group (Figure 5L). This means that we can select the appropriate checkpoint inhibitors and drugs for patients with different risk values according to the NRlncRNAs signature.

## Discussion

Osteosarcoma is a primary malignant tumor of bone in adolescents, which originates from mesenchymal tissue. Currently, the main treatment for osteosarcoma is neoadjuvant chemotherapy and surgery. However, limited progress has been made in the last 30 years to improve survival outcomes for patients with osteosarcoma, particularly, and the survival rate of patients with metastasis is less than 20%. Identifying a specific and reliable prognostic marker for OS is essential in improving the prognosis. Many lncRNAs play regulatory roles in the development and progression of OS. Yan et al. demonstrated that the lncRNA CCAT2 acts as an oncogene in osteosarcoma, promoting osteosarcoma cell proliferation, cell cycle, and invasion (Yan et al., 2018). Zheng et al. suggested that the lncRNA SNHG3 regulates osteosarcoma invasion and migration through the miRNA-151a-3p/RAB22A axis (Zheng et al., 2019).

Necroptosis has been shown to play an important role in the progression of many tumors, including osteosarcoma. Xiao et al. reported that graphene oxide–associated anti-HER2 antibodies have the ability to kill osteosarcoma cells *via* the necroptotic pathway (Xiao et al., 2019). Li et al. demonstrated that the nano-drug delivery system modified by polypeptide nanomaterials could kill osteosarcoma cells *in vitro* by inducing RIP1- and RIP3-dependent necroptosis (Li et al., 2018). After several studies asserted that necroptosis plays an important role in tumors, but the mechanisms involved are still not fully understood. Herein, to investigate the correlation between the tumor microenvironment, immune cell infiltration, immune checkpoints, and necroptosis-associated lncRNAs, this study constructed an NRlncRNA signature in patients with OS for the first time.

In this study, we initially obtained nine differentially expressed and prognostically relevant NRlncRNAs (AC124798.1, EPB41L4A-AS1, LINC01549, LINC01060, AP000851.2, SNHG1, AL354919.2, PVT1, and AL391121.1). Among these lncRNAs, SNHG1 promotes osteosarcoma progression *via* miR-493-5p as an oncogenic factor (Liu et al., 2022). PVT1 promotes osteosarcoma metastasis *via* miR-484 (Yan et al., 2020). Subsequently, we used three NRlncRNAs (AL391121.1, AL354919.2, and AP000851.2) to model risk through random grouping, univariate Cox regression score, and lasso regression analysis.

Apparently, AL391121.1 and AL354919.2 are positively regulated by TRIM11 and CD40, respectively. In addition, Wang et al. reported that TRIM11 was an oncogene gene in the growth of OS cells (Wang et al., 2019). CD40 plays an important role in tumor immunotherapy (Elgueta et al., 2009). In other words, TRIM11 may play a carcinogenic role through AL391121.1 and AL354919.2 may be involved in CD40-mediated immunotherapy. These lie just in line with the risk scoring formula. Then, based on the median risk score, patients were divided into a high-risk group and a low-risk group. The results showed that the low-risk group had better prognosis than the high-risk group and that the risk score was an independent predictor of OS patients. Similarly, the creation of predictive nomograms including clinicopathological variables and risk scores showed perfect agreement between observed and predicted rates for 1-, 3-, and 5-year overall survival.

Researchers demonstrated that necroptosis and lncRNAs are closely associated with tumorigenesis, tumor immune response, and prognosis, but the specific roles in these processes remain unclear (Xiao et al., 2019; Li and Wang, 2021). Therefore, we continued to explore potential mechanisms for the lncRNA signature associated with necroptosis between risk groups. GSEA showed that the high-risk group was significantly associated with the sphingolipid metabolism signaling pathway and the tight junction signaling pathway. The low-risk group was significantly associated with the primary immunodeficiency signaling pathway, hematopoietic cell lineage signaling pathway, B cell receptor signaling pathway, antigen processing and presentation signaling pathway, and natural killer cell pathway. Cortini et al. found that inhibition of the sphingolipid pathway impaired the survival and migration of osteosarcoma cells (Cortini et al., 2021). In addition, claudin1, as a tight junction protein, is increased in metastatic OS cells compared to primary tumor cells (Jian et al., 2015).

Due to the abundance of immune-related pathways in the low-risk population, we used ssGSEA to explore the immune status between risk groups. Immune cells (macrophages, neutrophils, T helper cells, and tumor infiltrating lymphocytes) and immune function (cytolytic activity) were predominantly active in the low-risk group. We also found that T cell CD4 memory activation and plasma cells were negatively associated with risk scores. In previous reports, macrophages and neutrophils were able to induce RIPK1-, RIPK3-, and MLKL-mediated necroptosis in cells (Shi et al., 2019; Wang et al., 2020). These results further suggest that necroptosis may be involved in the progression of OS by regulating tumor immunity. Although most reported immune checkpoints could help cancer cells evade immune destruction, some researchers claim that the expression levels of immune checkpoints positively correlate with the efficacy of immunotherapy (Marin-Acevedo et al., 2021; Lu et al., 2022). Therefore, we analyzed the correlation between the expression level of the immune checkpoint and NRlncRNA signature. Our results showed that the majority of immune checkpoint expression was elevated in patients in the low-risk group compared to those in the high-risk group. Kong et al. demonstrated that inhibiting the expression of CD44 can inhibit proliferation, migration, and invasion of osteosarcoma cells (Kong et al., 2022). Zhang et al. verified that the over-expression of LAIR-1 inhibited epithelial-mesenchymal transition in osteosarcoma (Jinxue Zhang et al., 2020). Thus, immunotherapy would be more beneficial for patients at a low risk of OS. Finally, we predicted some potentially suitable drugs for high-risk or low-risk patients, which may be useful for future treatment.

However, there are some limitations and shortcomings in our study. First, the potential mechanisms of NRlncRNAs in OS still need to be explored. Next, although we had internally validated by the whole group, training group, and testing group, external validation was not performed by other data.

In summary, the NRncRNA, as an independently prognostic marker of OS, could help forecast the procession of OS and provide guidance on immunotherapy for OS, but further validation is still needed.

## Data Availability

The datasets presented in this study can be found in online repositories. The names of the repository/repositories and accession number(s) can be found below: http://xena.ucsc.edu/.
